# 

**Published:** 1890-10

**Authors:** 


PHILOSOPHY IN HOMEOPATHY, addressed to the Medical Profession and to the General Reader. By CHARLES S. MACK, M. D., Professor of Materia Medica and Therapeutics in the Homeopathic Medical College of the University of Michigan at Ann Arbor. Chicago: Gross & Delbridge. 1890. Pp. 174; cloth, 8vo.

The author of this book looks on one side of the shield and tells us just what he sees from his standpoint. There is an earnestness in his style which we wish was devoted to matter which carried conviction.

But he is too firm a believer to convince. He is one of the few men who elevates a human system to a Divine law. He says: "I believe that homeopathy is without flaw." Believing this, we do not see how he could very well be anything but a homeopathist.

The publishers' work is well done. J. W. P.

